# In squamous cell carcinoma of the vulva, overexpression of p53 is a late event and neither p53 nor mdm2 expression is a useful marker to predict lymph node metastases

**DOI:** 10.1038/sj.bjc.6690318

**Published:** 1999-04

**Authors:** A G Emanuels, J Koudstaal, M P M Burger, H Hollema

**Affiliations:** Departments of Obstetrics and Gynecology and Pathology, University Hospital Groningen (AZG), Groniger, The Netherlands

**Keywords:** vulvar carcinoma, p53, mdm2, metastasis

## Abstract

To offer more tailored treatment to individual patients with squamous cell carcinoma of the vulva, more accurate prediction of lymph node metastases is required. As *p53* and *mdm2* are genes known to be involved in the development of other tumours, we studied expression of *p53* and *mdm2* in carcinogenesis of squamous cell carcinoma of the vulva and their clinical relevance. Archival material of 141 T1 and T2 vulvar tumours were used. Of the 141 primary tumours, the corresponding 39 lymph node metastases (LNM) were studied, and in 90 cases the pre-existent epithelia adjacent to the tumour (EAT) and in 14 cases vulvar intraepithelial neoplasia adjacent to the tumour (VIN) was also investigated. Detection of p53 and mdm2 protein was immunohistochemically performed. Scoring categories were: negative (1); weakly positive (2); moderately to markedly positive (3); and markedly positive (4). Overexpression of p53 was seen in 56% of the LNM, 39% of the primary tumours, 21% of the VIN lesions and 0% in the group of EAT. No relation was found between overexpression of p53 in the primary tumour and LNM. Expression of mdm2 was seen in 14% of the primary tumours, of which four cases were marked positive. In the group of LNM no mdm2-positive staining was observed. In the group of EAT, 25% was mdm2-positive, of which six cases were marked positive. In the group of VIN, 36% showed moderate (score 3) mdm2 expression. No relation was found between expression of mdm2 and LNM. In squamous cell carcinoma, overexpression of p53 is a late event in carcinogenesis. Marked expression of mdm2 is rarely seen in vulvar carcinomas, indicating that aberrant p53 cannot induce mdm2 expression. LNM cannot be predicted by detection of these proteins. © 1999 Cancer Research Campaign


*British Journal of Cancer* (1999) **80**(1/2), 38–43
© 1999 Cancer Research Campaign
Article no. bjoc.1998.0318

### In squamous cell carcinoma of the vulva, overexpression of p53 is a late event and neither p53 nor mdm2 expression is a useful marker to predict lymph node metastases

**AG Emanuels[1], J Koudstaal[2], MPM Burger[2] and H Hollema[2]**

Departments of [1]Obstetrics and Gynecology and [2]Pathology, University Hospital Groningen (AZG), Groniger, The Netherlands

**Summary** To offer more tailored treatment to individual patients with squamous cell carcinoma of the vulva, more accurate prediction of lymph node metastases is required. As *p53* and *mdm2* are genes known to be involved in the development of other tumours, we studied expression of *p53* and *mdm2* in carcinogenesis of squamous cell carcinoma of the vulva and their clinical relevance. Archival material of 141 T1 and T2 vulvar tumours were used. Of the 141 primary tumours, the corresponding 39 lymph node metastases (LNM) were studied, and in 90 cases the pre-existent epithelia adjacent to the tumour (EAT) and in 14 cases vulvar intraepithelial neoplasia adjacent to the tumour (VIN) was also investigated. Detection of p53 and mdm2 protein was immunohistochemically performed. Scoring categories were: negative (1); weakly positive (2); moderately to markedly positive (3); and markedly positive (4). Overexpression of p53 was seen in 56% of the LNM, 39% of the primary tumours, 21% of the VIN lesions and 0% in the group of EAT. No relation was found between overexpression of p53 in the primary tumour and LNM. Expression of mdm2 was seen in 14% of the primary tumours, of which four cases were marked positive. In the group of LNM no mdm2-positive staining was observed. In the group of EAT, 25% was mdm2-positive, of which six cases were marked positive. In the group of VIN, 36% showed moderate (score 3) mdm2 expression. No relation was found between expression of mdm2 and LNM. In squamous cell carcinoma, overexpression of p53 is a late event in carcinogenesis. Marked expression of mdm2 is rarely seen in vulvar carcinomas, indicating that aberrant p53 cannot induce mdm2 expression. LNM cannot be predicted by detection of these proteins.

**Keywords:** vulvar carcinoma; p53; mdm2; metastasis

For patients with squamous cell carcinoma of the vulva, surgical therapy comprises vulvectomy or wide local excision and bilateral or unilateral lymphadenectomy. Better insight into carcinogenesis and progression of the disease is needed to provide arguments for a more tailored treatment of the individual patient. As *p53* and *mdm2* are genes known to be involved in development of other tumours, we investigated the presence of *p53* and *mdm2* in carcinogenesis of squamous cell carcinoma of the vulva and whether immunohistochemical detection of the gene products is of clinical relevance.

*p53*, a tumour suppressor gene located on the short arm of chromosome 17, plays an important role in the regulation of the cell cycle (Hartwell and Kasten, 1994; Prokocimer and Rotter, 1994). Genetic alteration of this gene is associated with prognostic relevance in several tumours (Charpin et al, 1995; Esrig et al, 1994; Florenes et al, 1994; Shurbaji et al, 1995; Sun et al, 1992; Vogt et al, 1997), including vulvar carcinomas (Kohlberger et al, 1995; Milde-Langosch et al, 1995). It has been shown that cells defective for the *p53* gene continue to enter the S phase after irradiation with an increased chance for aberrant DNA to be duplicated. Loss of the G1–S checkpoint in cell division can lead to genomic instability and potential development of malignancy. *p53* is presumed to prevent genomic instability, upon exposure to DNA damaging agents (Lane, 1992).

Mutation of the *p53* gene gives rise to a p53 oncoprotein that is more stable than the wild-type (wt) protein and therefore can accumulate in the cell. Detection of the mutant p53 protein is possible by immunohistochemistry (Lassam et al, 1993; Shurbaji et al, 1995). However, non-sense mutations, or mutations not encoded on exon 5 to exon 8 of the *p53* gene, are not detectable on protein level (Bosari and Viale, 1995; Bosari et al, 1995).

The *mdm2* gene, localized on chromosome 12q13–14, is presumed to be a negative regulator of *p53*. This is based on the finding that *mdm2* gene product, a 95 kDa protein, can form complexes with the p53 protein, and overexpression of *mdm2* inhibits the functioning of p53 (Momand et al, 1992). Moreover, amplification of *mdm2* is shown to be involved in tumorigenesis of human sarcomas (Oliner et al, 1992). Furthermore it is demonstrated that expression of the *mdm2* gene is regulated by transient induction of wt p53 activity (Barak et al, 1993). Wu et al (1993) demonstrated that the induction of *mdm2* by p53 occurs at the level of transcription.

The aims of this study of squamous cell carcinoma of the vulva were: to establish the pattern of expression of p53 and mdm2 protein in primary tumours, vulvar intraepithelial neoplasia (VIN), epithelia adjacent to the tumour (EAT) and in lymph node metastases (LNM); to determine whether *p53* and *mdm2* are involved

*Received 19 December 1997*
*Revised 28 September 1998*
*Accepted 20 October 1998*

*Correspondence to:* A G Emanuels, Lombardi Cancer Center, Georgetown University, E304, New Research Building, 3950 Reservoir Road, Washington, DC 20007, USA

in metastasis; and to address the possible relationship between *p53* and *mdm2* expression.

### MATERIALS AND METHODS

### Patients

Data were obtained from samples of 141 patients with primary invasive squamous cell carcinoma of the vulva who were treated with vulvectomy (115) or wide local excision (26) and bilateral inguinofemoral lymphadenectomy. None of these patients received preoperative therapy. All patients were surgically treated between 1982 and 1992 at the Department of Gynecological Oncology, University Hospital Groningen. The tumours did not extend to the urethra, vagina or anus and were not fixed to the pelvis (T1 and T2 tumours). Depth of invasion was more than 1 mm. The age range of the patients was 29–94 years with a median value of 71 years. Twenty-eight per cent of the patients (39/141) had inguinofemoral LNM. Pre-existent EAT of 90 patients and VIN lesion adjacent to the tumour of 14 patients were also studied. EAT with morphologic abnormalities consistent with known disease entities, such as lichen sclerosis and hyperplasia, were not included. This series consisted of 64 patients with differentiation grade 1, 63 with grade 2 and 14 with grade 3.

### Methods

#### *mdm2* and *p53* immunostaining

We used formalin-fixed, paraffin-embedded tissue of vulvar tumours. Sections of 4 μm were mounted on APES-coated slides (amino-propyl-ethoxy-silan; SIGMA), deparaffinized, rehydrated to 96% alcohol and air dried. For antigen retrieval we used an autoclave (Emanuels et al, 1994) in which slides were heated three times 5 min at 115°C in blocking reagent (Boehringer Mannheim) [2% block + 0.2% sodium dodecyl sulphate (SDS) in maleic acid, pH = 6.0]. After antigen retrieval, one series of slides was incubated with Bp53–12 (80 × diluted), which is a monoclonal antibody recognizing wt and mutant-type p53 protein (BioGenex, San Ramon, CA, USA) and another series of slides was incubated with mdm2 Ab-1 (100 × diluted), which is a mouse monoclonal against human mdm2 protein (Oncogene Science, Uniondale, NY, USA). Two-step immunostaining was performed according to the manufacturer's procedure of the Biogenex kit, containing anti-mouse biotin and conjugated streptavidin. BCIP-NBT (bromochloro-indolyl-phosphate 4-nitroblue-tetrazolium chloride; Boehringer Mannheim) was used as substrate. Sections were counterstained with haematoxylin and mounted with mounting medium. As negative control, IgG2a was used instead of p53, IgG2b instead of mdm2. As a positive control multi-tissue block sections were used composed of 24 different vulvar carcinomas (control for p53) and one normal skin (control for mdm2). p53 and mdm2 were semi-quantitatively scored. The scoring categories were: negative (1), weakly positive (2), moderately to markedly positive (3) and markedly positive (4). Scores 1 and 2 were grouped together and considered non-expression, and scores 3 and 4 were grouped together and considered expression.

### Statistical analysis

Pearson $\chi^2$ test was used to compare two categorical variables. Statistical analysis was performed with computer software of the statistical program SYSTAT.

### RESULTS

The group of 141 primary vulvar carcinomas was divided in 118 large primary tumours (invasion depth ≥ 3 mm) and 23 small primary tumours (invasion depth < 3 mm) (Chu et al, 1982). Of the 141 primary tumours, the corresponding 39 LNM, 90 cases of pre-existent EAT and 14 cases of VIN lesions adjacent to the tumour were investigated.

Table 1 represents the scoring categories based on a combination of staining intensity and pattern. Staining intensity of the p53-positive cells varied between the different cases, ranging from weakly to moderately to markedly positive. Staining pattern was defined as absent, patchy and diffuse. Heterogeneous staining was seen in category 3. Overexpression (score 4) of p53 (Figure 1) was seen in 56% of the LNM, 39% of the large tumours, 39% of the small tumours and 21% of the VIN. The group of EAT was p53-negative.

Table 2 represents the distribution of p53 protein expression in LNM, large tumours, small tumours, VIN lesions and EAT. p53 was seen in undifferentiated non-keratinizing cells. In all cases, p53 staining was restricted to dysplastic or tumour cells.

Figure 2 shows the distribution of p53 in the groups studied. An increase in p53 staining was seen from the group of VIN to the group of metastases. Marked p53 staining was predominantly seen in LNM, large and small tumours, and less frequently in the group of VIN lesions. No p53-positive cases were found in the group of EAT.

In the groups investigated no significant relation was found between p53 expression and the absence or presence of LNM. Neither was there a significant relation with depth of invasion, differentiation grade, age and greatest diameter. A total of 35% (19/55) of the markedly positive primary tumours were metastasized. Expression of p53 in the 39 metastases was highly correlated with expression of p53 in the corresponding primary tumours

**Table 1** Categories of p53/mdm2 scoring

| Score | Staining intensity | Staining pattern |
|---|---|---|
| 1 | Negative | Absent |
| 2 | Weak | Patchy |
| 3 | Moderate and marked | Patchy |
| 4 | Marked | Diffuse |

**Table 2** Number and percentage of p53 expression in the group of metastases, large vulvar tumours, small vulvar tumours, VIN and EAT

| p53 | 1 | 2 | 3 | 4 | Total |
|---|---|---|---|---|---|
| Metastases | 0 | 14 | 3 | 22 | 39 |
| | 0% | 36% | 8% | 56% | 100% |
| Large tumours | 39 | 20 | 13 | 46 | 118 |
| | 33% | 17% | 11% | 39% | 100% |
| Small tumours | 5 | 4 | 5 | 9 | 23 |
| | 22% | 17% | 22% | 39% | 100% |
| VIN | 9 | 2 | – | 3 | 14 |
| | 65% | 14% | – | 21% | 100% |
| EAT | 90 | – | – | – | 90 |
| | 100% | – | – | – | 100% |

VIN = vulvar intraepithelial neoplasia adjacent to tumour; EAT = epithelial adjacent to the tumour

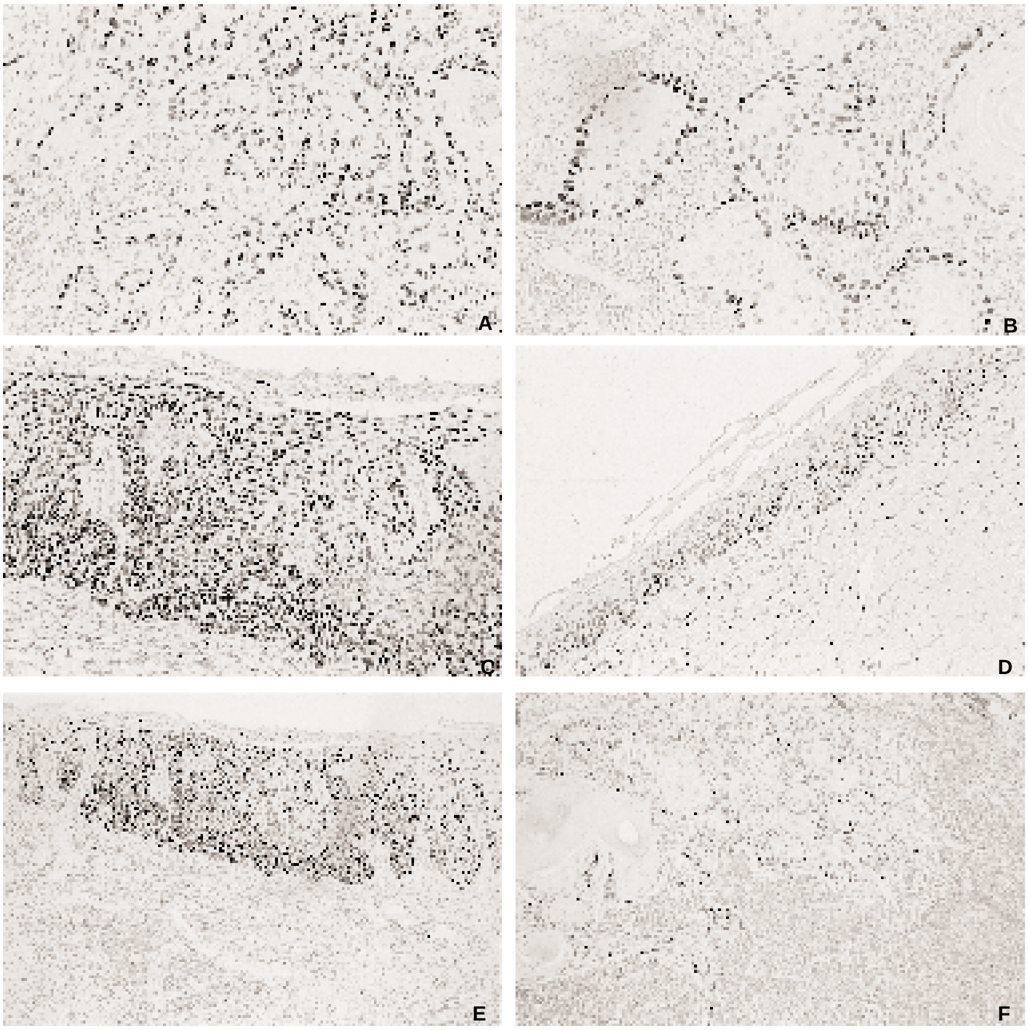

**Figure 1** Overexpression of p53 in the primary tumour (**A** and **B**), in VIN (**C** and **D**) (**E** is an overview of **C**) and in a LNM (**F**)

(Pearson $\chi^2$; $P$ = 0.001). Of the 16 markedly p53-positive metastatic primary tumours, 15 corresponding LNM were also markedly positive and one was weakly positive. No p53-negative LNM were found.

In this study, mdm2 staining pattern was comparable to that of p53 and the same scoring system was used (Table 1). mdm2-positive cells were localized in the basal layer of EAT and diffusely throughout VIN lesions and tumours, except for keratin pearls. In the group of metastases only weak staining was seen (Figure 3). In general, expression of mdm2 protein was seen less frequently (20/141) compared to p53 staining (63/141). mdm2 expression tended to decline from EAT to VIN to primary tumours (Figure 4).

Table 3 shows the distribution of expression of mdm2 in the groups of LNM, large tumours, small tumours, VIN lesions and EAT. The highest percentage of mdm2-positive cases were found in the group of VIN lesions (36%) and EAT (25%), declining in the group of small tumours (22%) and large tumours (12%). Four diffuse markedly positive cases were found in the group of large tumours (which were the only four diffuse markedly positive cases found in the total group of primary tumours). The number of mdm2-positive cases in the primary tumours was too low to establish a statistically significant relation between expression of mdm2 and other tumour parameters.

In EAT expression, p53 was absent whereas mdm2 was present and the opposite was seen in the group of metastases, showing only slight expression of mdm2 and distinct expression of p53. Co-expression of p53 and mdm2 was found in 6.5% (moderate to marked staining) and in 7% (weak staining) of the primary tumours. Inverse expression was found in 53% of the primary tumours. Within the groups investigated, no significant relation was found between expression of p53 and mdm2.

### DISCUSSION

To individualize surgical therapy for low-risk patients with vulvar carcinoma, additional parameters to predict LNM are needed. We investigated expression of p53 and mdm2 protein in relation to LNM and clincopathological parameters, in a series of 141 T1/T2 squamous cell carcinomas of the vulva, to assess clinical relevance. We were also interested in the distribution and staining pattern of both gene products and a possible relationship between p53 and mdm2 and their involvement in carcinogenesis of squamous cell carcinoma of the vulva.

 *© Cancer Research Campaign 1999*

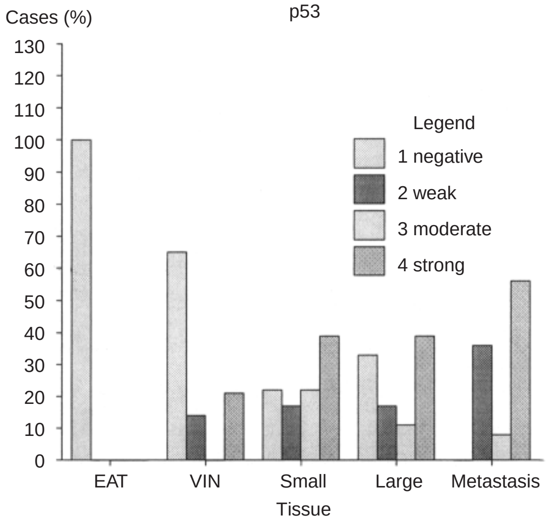

**Figure 2** Distribution of p53 expression in the groups of EAT, VIN, small tumours, large tumours and LNM

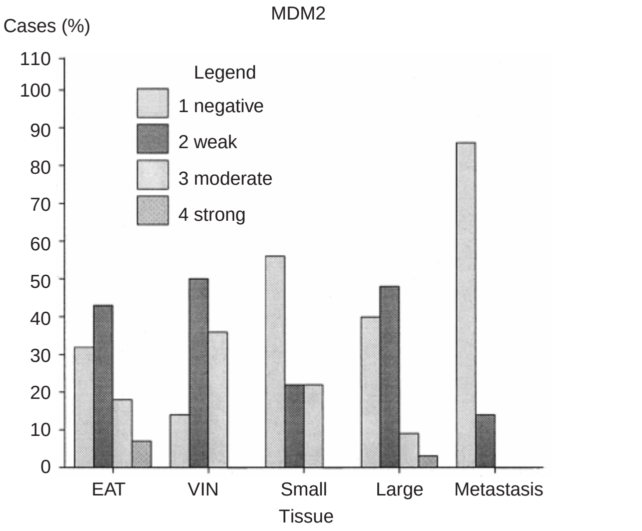

**Figure 4** Distribution of mdm2 expression in the groups of EAT, VIN, small tumours, large tumours and LNM

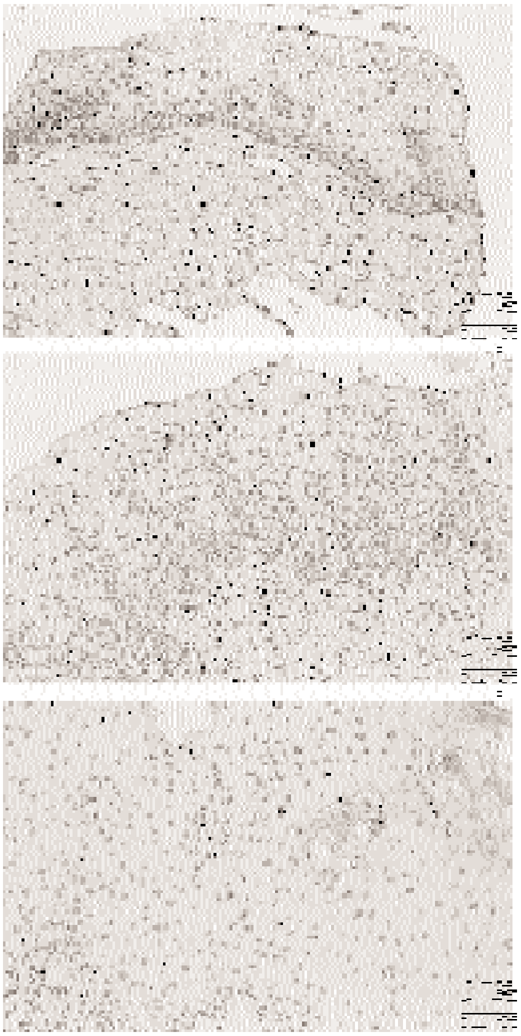

**Figure 3** mdm2 staining in EAT (**A**), in VIN (**B**) and in the primary tumour (**C**)

**Table 3** Number and percentage of mdm2 expression in the group of metastases, large tumours, small tumours, VIN and EAT

| mdm2 | 1 | 2 | 3 | 4 | Total |
|---|---|---|---|---|---|
| Metastasis | 32 | 5 | 0 | 0 | 37 |
| | 86% | 14% | 0% | 0% | 100% |
| Large tumours | 47 | 56 | 11 | 4 | 118 |
| | 40% | 48% | 9% | 3% | 100% |
| Small tumours | 13 | 5 | 5 | 0 | 23 |
| | 56% | 22% | 22% | 0% | 100% |
| VIN | 2 | 7 | 5 | – | 14 |
| | 14% | 50% | 36% | – | 100% |
| EAT | 29 | 39 | 16 | 6 | 90 |
| | 32% | 43% | 18% | 7% | 100% |

VIN = vulvar intraepithelial neoplasia adjacent to tumour; EAT = epithelial adjacent to the tumour.

Previous studies have shown that enhanced expression of p53 is related to mutation in the *p53* gene (Bosari et al, 1995; Esrig et al, 1993; Przygodzki et al, 1996), but this is contradicted by others (Kennedy et al, 1994; Marchetti et al, 1995). In our study we detected overexpression of p53 protein and found a higher percentage of strongly positive tumours in the malignant versus the premalignant group (Figure 2). Whether overexpression of p53 in this study reflects a mutation or not, our data indicate that enhanced p53 staining is related to progression from the precursor lesion to the ultimate development of metastases.

In our study of vulvar tumours, immunohistochemical detection of p53 overexpression did not contribute to the prediction of LNM. Neither have we found a relation between p53 and clinicopathological parameters age, depth of invasion and differentiation grade. In their study of vulvar carcinomas, Kagie et al (1997) did not find a relationship between p53 overexpression and disease-free survival. Kohlberger et al (1995) have reported a relationship between p53 overexpression and survival in vulvar carcinomas, based on a group of 25 vulvar carcinomas. In the study by Milde-Langosch et al, (1995) on vulvar cancer, loss of p53 function was

*© Cancer Research Campaign 1999* 

associated with a high risk of progression and an unfavourable prognosis. In the literature the prognostic value of p53 seems to be controversial, because in some studies p53 is considered prognostic relevant (Charpin et al, 1995; Esrig et al, 1994; Florenes et al, 1994; Shurbaji et al, 1995; Sun et al, 1992; Vogt et al, 1997), whereas in other studies it is not (Bosari et al, 1995; King et al, 1996; Ofner et al, 1995; Xerri et al, 1994; Younes et al, 1995). It may be that the prognostic value of p53 depends on whether mutation of p53 occurs during carcinogenesis, progression or metastasis of the tumour. Moreover, based on our finding that only 35% of the enhanced p53-positive cases were metastasized it seems that additional oncogenic events are necessary for the development of LNM in vulvar carcinomas.

We divided the group of primary tumours in large (invasion depth > 3 mm) and small (invasion depth ≤ 3 mm) tumours (Chu et al, 1982), to see whether the occurrence of aberrant expression of p53 is relatively higher in large tumours than in small tumours. No difference was found between the groups of small and large tumours with respect of expression of p53, indicating that once a tumour has been developed, aberrant expression of p53 can occur regardless of the depth of invasion of the tumour.

p53 overexpression can occur early or late in carcinogenesis, depending on the tumour type (Charpin et al, 1995; Conlter et al, 1995; Kennedy et al, 1994; Lassam et al, 1993). Overexpression of p53 in our series of vulvar carcinomas appears to occur predominantly in LNM and primary tumours, and to a much lesser extent in VIN lesions, and p53 expression is absent in morphologic normal EAT. This may indicate that overexpression of p53 is a late event in the development of squamous cell carcinoma of the vulva, playing a role in tumour progression. Heterogeneity in p53 staining intensity within a tumour was found in 13% of the primary tumours. It is suggested by Esrig et al (1993) that this seems to be related to the site of the mutation of the *p53* gene or a combination of wt and mutant-type expressed p53. Weak staining could represent overexpression of wt p53. LNM and the corresponding primary tumours show similar patterns of p53 expression, strongly indicating that in metastasized cells the same aberrant p53 is expressed as in the primary tumour. This was also reported by Florenes et al (1994), who found in four out of five p53-positive primary tumours, the same degree of positive immunostaining in the corresponding metastases. Overexpression of p53 protein seems to favour survival of metastatic tumour cells.

The reported incidence of *mdm2* gene amplification is 10–36% in non-epithelial tumours, whereas reports regarding epithelial tumours showed no evidence of aberrant *mdm2* gene copy number (McCann et al, 1995). In the group of primary tumours we found 14% (20/141) mdm2-enhanced positive cases of which only 4/141 were diffuse markedly positive. Our data show that enhanced expression of mdm2 is a rare event in vulvar carcinomas, which is also found in other studies of epithelial tumours (Kessis et al, 1993; Marchetti et al, 1995; Quesnel et al, 1994).

No relationship was found between the expression of mdm2 protein and LNM. Whether immunohistochemical detection of mdm2 expression in this study represents amplification of the gene is open to discussion. We found no mdm2 staining in LNM and rarely in the primary tumour in contrast to EAT and VIN. This is in accordance to the study of Dazard et al (1997), who found that mdm2 is expressed in normal skin, with lower levels of expression in squamous cell carcinoma. It seems likely that the presence of mdm2 staining in the EAT represents normal expression of the protein. Though mdm2 amplification is rarely seen in epithelial tumours, it would be interesting to perform genomic analysis on the four primary tumours and the six EATs showing strong expression of mdm2.

In recent models regarding interaction of tumour suppressor genes and proto-oncogenes, mdm2 is designated as a regulator of the *p53* gene and the mdm2 feedback mechanism is activated by wild-type p53 (Barak et al, 1993). In this regard it would be expected that overexpression of mutant p53 goes together with low, or no, expression of mdm2. Within the group of primary tumours, we found 13% co-expression of p53 and mdm2, and 53% inverse expression, but a relationship could not be statistically established. However, we found an increasing percentage of p53-positive cases in the range of EAT, VIN, primary tumours and metastases. Exactly the opposite is found for mdm2 expression, which shows a decreasing percentage of mdm2-positive cases in the same range. These data suggest that aberrant p53 does not activate mdm2 expression.

We investigated VIN lesions and pre-existent EAT hypothesizing that these non-malignant cells are not as much dysregulated as the tumour cells and that detection of p53 and mdm2 protein in these cells could be used as a possible marker of dysregulation of these genes in the tumour cells. No such relationship was found.

We conclude from this study that, in squamous cell carcinoma of the vulva, overexpression of p53 protein is a late event and that overexpression of p53, even though not genotypically confirmed, reflects abnormality. Strong expression of mdm2 is rarely seen in vulvar tumours, indicating that, in squamous cell carcinoma of the vulva, aberrant p53 cannot induce mdm2. Expression of p53 or mdm2 cannot be used to predict lymph node metastases.

### REFERENCES

Barak Y, Juven T, Haffner R and Oren M (1993) mdm2 expression is induced by wild type p53 activity. *EMBO J* **12**: 461–468

Bosari S and Viale G (1995) The clinical significance of p53 aberrations in human tumors. *Virchows Arch* **427**: 229–241

Bosari S, Viale G, Roncalli M, Graziani D, Borsani G, Lee AK and Coggi G (1995) p53 gene mutations, p53 protein accumulation and compartmentalization in colorectal adenocarcinoma. *Am J Pathol* **147**: 790–798

Charpin C, Devictor B, Andrac L, Amabile J, Bergeret D, Allasia C and Piana L and LaVaut MN (1995) p53 quantitative immunocytochemical analysis in breast carcinomas. *Hum Pathol* **26**: 159–166

Chu J, Tamimi H, Ek M and Figge D (1982) Stage I vulvar cancer: criteria for microinvasion. *Obstet Gynecol* **59**: 716–719

Coulter LK, Wolber R and Tron VA (1995) Site-specific comparison of p53 immunostaining in squamous cell carcinomas. *Hum Pathol* **26**: 531–533

Dazard J, Augias D, Neel H, Mils V, Marechal V, Basset-Seguin N and Piette J (1997) Mdm2 protein is expressed in different layers of normal human skin. *Oncogene* **14**: 1123–1128

Emanuels A, Hollema H, Suurmeyer A and Koudstaal J (1994) A modified method for antigen retrieval MIB-1 staining of vulvar carcinoma. *Eur J Morphol* **32**: 335–337

Esrig D, Elmajian D, Groshen S, Freeman JA, Stein JP, Chen SC, Nichols PW, Skinner DG, Jones PA and Cote RJ (1994) Accumulation of nuclear p53 and tumor progression in bladder cancer [see comments]. *N Engl J Med* **331**: 1259–1264

Esrig D, Spruck CH, Nichols PW, Chaiwun B, Steven K, Groshen S, Chen SC, Skinner DG, Jones PA and Cote RJ (1993) p53 nuclear protein accumulation correlates with mutations in the p53 gene, tumor grade, and stage in bladder cancer. *Am J Pathol* **143**: 1389–1397

Florenes VA, Oyjord T, Holm R, Skrede M, Borresen AL, Nesland JM and Fodstad O (1994) TP53 allele loss, mutations and expression in malignant melanoma. *Br J Cancer* **69**: 253–259

Hartwell LH and Kastan MB (1994) Cell cycle control and cancer. *Science* **266**: 1821–1828

Kagie M, Kenter G, Tollenaar R, Hermans J, Baptist Trimbos J and Fleuren G (1997) p53 protein overexpression is common and independent of human

© Cancer Research Campaign 1999

papillomavirus infection in squamous cell carcinoma of the vulva. *Cancer* **80**: 1228–1233

Kennedy SM, Macgeogh C, Jaffe R and Spurr NK (1994) Overexpression of the oncoprotein p53 in primary hepatic tumors of childhood does not correlate with gene mutations [see comments]. *Hum Pathol* **25**: 438–442

Kessis TD, Slebos RJ, Han SM, Shah K, Bosch XF, Munoz N, Hedrick L and Cho KR (1993) p53 gene mutations and MDM2 amplification are uncommon in primary carcinomas of the uterine cervix. *Am J Pathol* **143**: 1398–1405

King LA, Okagaki T, Gallup DG, Twiggs LB, Messing MJ and Carson LF (1996) Mitotic count, nuclear atypia, and immunohistochemical determination of Ki-67, c-myc, p21-ras, c-erbB2, and p53 expression in granulosa cell tumors of the ovary: mitotic count and Ki-67 are indicators of poor prognosis. *Gynecol Oncol* **61**: 227–232

Kohlberger P, Kainz C, Breitenecker G, Gitsch G, Sliutz G, Kolbl H, Tschachler E and Reinthaller A Prognostic value of immunohistochemically detected p53 expression in vulvar carcinoma. *Cancer* **76**: 1786–1789

Lane DP (1992) p53, guardian of the genome. *Nature* **358**: 15–16

Lassam NJ, From L and Kahn HJ (1993) Overexpression of p53 is a late event in the development of malignant melanoma. *Cancer Res* **53**: 2235–2238

Marchetti A, Buttitta F, Pellegrini S, Merlo G, Chella A, Angeletti CA and Bevilacqua G (1995) mdm2 gene amplification and overexpression in non-small cell lung carcinomas with accumulation of the p53 protein in the absence of p53 gene mutations. *Diagn Mol Pathol* **4**: 93–97

McCann AH, Kirley A, Carney DN, Corbally N, Magee HM, Keating G and Dervan PA (1995) Amplification of the MDM2 gene in human breast cancer and its association with MDM2 and p53 protein status. *Br J Cancer* **71**: 981–985

Milde-Langosch K, Albrecht K, Joram S, Schlechte H, Giessing M and Loning T (1995) Presence and persistence of HPV infection and p53 mutation in cancer of the cervix and the vulva. *Int J Cancer* **63**: 639–645

Momand J, Zambetti GP, Olson DC, George D and Levine AJ (1992) The mdm-2 oncogene product forms a complex with the p53 protein and inhibits p53-mediated transactivation. *Cell* **69**: 1237–1245

Ofner D, Maier H, Riedmann B, Holzberger P, Nogler M, Totsch M, Bankfalvi A, Winde G, Bocker W and Schmid K (1995) Immunohistochemically detectable p53 and mdm-2 oncoprotein expression in colorectal carcinoma: prognostic significance. *J Clin Pathol Mol Pathol* **48**: 12–16

Oliner JD, Kinzler KW, Meltzer PS, George DL and Vogelstein B (1992) Amplification of a gene encoding a p53-associated protein in human sarcomas [see comments]. *Nature* **358**: 80–83

Prokocimer M and Rotter V (1994) Structure and function of p53 in normal cells and their aberrations in cancer cells: projection on the hematologic cell lineages. *Blood* **84**: 2391–2411

Przygodzki RM, Finkelstein SD, Langer JC, Swalsky PA, Fishback N, Bakker A, Guinee DG, Koss M and Travis WD (1996) Analysis of p53, K-ras-2, and C-raf-1 in pulmonary neuroendocrine tumors. Correlation with histological subtype and clinical outcome. *Am J Pathol* **148**: 1531–1541

Quesnel B, Preudhomme C, Fournier J, Fenaux P and Peyrat JP, (1994) MDM2 gene amplification in human breast cancer. *Eur J Cancer* **30A**: 982–984

Shurbaji MS, Kalbfleisch JH and Thurmond TS (1995) Immunohistochemical detection of p53 protein as a prognostic indicator in prostate cancer. *Hum Pathol* **26**: 106–109

Sun XF, Carstensen JM, Zhang H, Stal O, Wingren S, Hatschek T and Nordenskjold B (1992) Prognostic significance of cytoplasmic p53 oncoprotein in colorectal adenocarcinoma. *Lancet* **340**: 1369–1373

Vogt T, Zipperer KH, Vogt A, Holzel D, Landthaler M and Stolz W (1997) p53-protein and Ki-67 antigen expression are both reliable biomarkers of prognosis in thick stage 1 nodular melanomas of the skin. *Histopathology* **30**: 57–63

Wu X, Bayle J, Olson D and Levine A (1993) The p53-mdm2 autoregulatory feedback loop. *Genes Dev* **7**: 1126–1132

Xerri L, Bouabdallah R, Camerlo J and Hassoun J (1994) Expression of the p53 gene in Hodgkin's disease: dissociation between immunohistochemistry and clinicopathological data. *Hum Pathol* **25**: 449–454

Younes M, Lebovitz RM, Bommer KE, Cagle PT, Morton D, Khan S and Laucirica R (1995) p53 accumulation in benign breast biopsy specimens. *Hum Pathol* **26**: 155–158

*© Cancer Research Campaign 1999*
